# Population genomics of the invasive Northern Giant Hornet *Vespa mandarinia* in North America and across its native range

**DOI:** 10.1038/s41598-024-61534-0

**Published:** 2024-05-11

**Authors:** Benjamin A. Taylor, Luke R. Tembrock, Madison Sankovitz, Telissa M. Wilson, Chris Looney, Junichi Takahashi, Todd M. Gilligan, Allan H. Smith-Pardo, Brock A. Harpur

**Affiliations:** 1 Department of Entomology, Purdue University, West Lafayette, IN 47907 USA; 2 Department of Agricultural Biology, Colorado State University, Fort Collins, CO 80523 USA; 3 Department of Ecology and Evolutionary Biology and BioFrontiers Institute, University of Colorado Boulder, Boulder, CO 80303 USA; 4 Washington State Department of Agriculture, Olympia, WA 98501 USA; 5 Faculty of Life Sciences, Kyoto Sangyo University, Kyoto, 603-8047 Japan; 6 USDA Animal and Plant Health Inspection Service (APHIS), Fort Collins, CO 80526-1825 USA

**Keywords:** Invasive species, Population genetics, Entomology

## Abstract

The northern giant hornet *Vespa mandarinia* (NGH) is a voracious predator of other insect species, including honey bees. NGH’s native range spans subtropical and temperate regions across much of east and southeast Asia and, in 2019, exotic populations of the species were discovered in North America. Despite this broad range and invasive potential, investigation of the population genomic structure of NGH across its native and introduced ranges has thus far been limited to a small number of mitochondrial samples. Here, we present analyses of genomic data from NGH individuals collected across the species’ native range and from exotic individuals collected in North America. We provide the first survey of whole-genome population variation for any hornet species, covering this species’ native and invasive ranges, and in doing so confirm likely origins in Japan and South Korea for the two introductions. We additionally show that, while this introduced population exhibited strongly elevated levels of inbreeding, these signatures of inbreeding are also present in some long-standing native populations, which may indicate that inbreeding depression alone is insufficient to prevent the persistence of NGH populations. As well as highlighting the importance of ongoing monitoring and eradication efforts to limit the spread of this species outside of its natural range, our data will serve as a foundational database for future genomic studies into introduced hornet populations.

## Introduction

The northern giant hornet (NGH) *Vespa mandarinia* Smith 1852 is widespread across much of southern and eastern Asia, with a distribution ranging from Hokkaido, Japan west to northern India and from far eastern Russia south to Hong Kong^1,2^. Like other hornets, this species forms annual eusocial colonies and predates upon other arthropods to provision developing larvae^3,4^. NGH is one of the largest two hornet species and is among the most important predators of honey bees across its endemic range^4,5^. The sympatric honey bee species *Apis cerana* possesses natural defenses against NGH, including specialised alarm signals^6,7^, collection and use of deterrent odorants^8^, and coordinated thermal defence (‘heat-balling’) against hornet attacks^9^. Because its natural range does not overlap with that of NGH, the western honey bee *Apis mellifera* lacks these specialised natural defences^6,10^ and, consequently, is highly vulnerable to attacks by this predator^5,11^.

Like other social insects, hornets have a high potential to become invasive^4,12,13^. At least five hornet species have successfully established populations outside of their native range^4^: *Vespa velutina* in Korea, Japan, and much of western Europe; *V. orientalis* in southern Spain and Gibraltar; *V. tropica* in Guam; *V. bicolor* in Taiwan and Spain; and *V. crabro* in eastern North America. However, genomic data are lacking for the majority of these invasive populations, especially at the earliest stages of introduction. To our knowledge, the only exceptions to this knowledge gap are two studies respectively using *V. mandarinia*^14^ and *V. velutina*^15^, both of which were restricted to a small number of samples and genetic loci. Better understanding the genetic correlates of these hornet introductions may provide insights into the likelihood that a newly introduced hornet population will become established^4^. In particular, because of the negative fitness effects of inbreeding depression, the amount of genetic diversity present in an introduced population may be a significant factor limiting the invasive potential of that population^16,17^. Factors such as polyandry and multiple founding, which increase the genetic diversity present in a single founding event, are therefore likely to contribute to invasion potential in hornets^4,15^.

**Figure 1:**
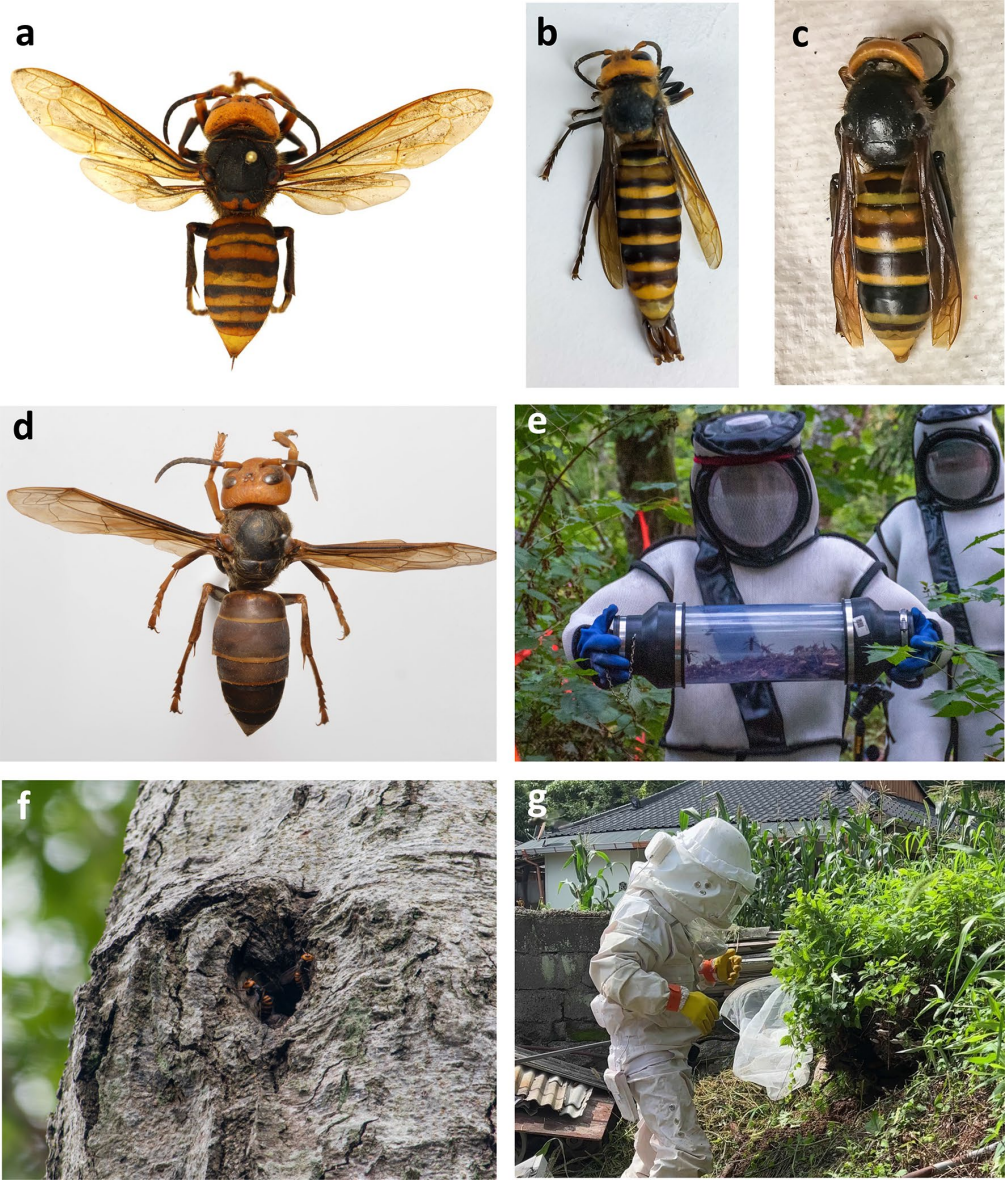
Selected photos documenting NGH collection and eradication efforts between 2019 and 2021. Dorsal views of (**a**) worker collected in Nanaimo, BC, September 2019; (**b**) male collected in Blaine, WA, July 2020; (**c**) worker collected in Birch Bay, WA, July 2020; (**d**) worker collected in Yunnan, China. (**e**) WSDA agents with samples collected during the removal of a nest in Blaine, WA, September 2021, and (**f**) NGH workers at the entrance of the same nest prior to removal. (**g**) Hornet hunter removing an NGH nest near a residence, South Korea.

In September 2019, an NGH nest was discovered and eradicated in Nanaimo, British Columbia, Canada^14,18^. In December of the same year, an NGH specimen was identified in Blaine, Washington, USA, approximately 95 km southeast of the nest found in Nanaimo^14^. The identification of these samples, together with the known vulnerability of agriculturally important *A. mellifera* honey bee nests to attacks by NGH, resulted in a concerted effort by agricultural agencies to track and eradicate this hornet species and plan for future introductions. Over the following two years, survey efforts by local government agencies and the public resulted in another 45 confirmed sightings and specimens in British Columbia and Washington, leading to the eradication of four colonies in Washington State (one in 2020 and three in 2021; Fig. 1a–e)^19^.

No further NGH were detected in Washington or mainland British Columbia in 2022, 2023, or at time of writing in 2024, nor were there any additional confirmed sightings in Nanaimo following the 2019 eradication. These results indicate that some combination of human eradication efforts, Allee effects^20,21^, ecological factors and chance have likely extirpated the incipient North American NGH populations identified in 2019. Nonetheless, hornets possess a high capacity for invasiveness^4^, and species distribution models indicate that much of North America may be climatically suitable for the persistence of NGH populations, including areas important for commercial honey production^22–24^. Moreover, analysis of mitochondrial data from two of the samples collected in 2019 indicated separate geographic origins for the Nanaimo and Washington introductions despite the geographic and temporal proximity of the samples^14^, indicating repeated introductions of the same invasive species within a short window of time. However, that study was based on mitochondrial data alone and included only a small number of individuals (whole mitogenomes for five samples and partial mitochondrial barcode sequences for an additional eight). Those data are therefore insufficient to precisely assess the degree of diversity within the surveyed populations, to estimate the degree to which those populations display signatures of inbreeding, or to address the precise localities from which the invasive introductions originated. A more thorough understanding of the route(s) by which NGH reached North America in 2019 and of the mechanisms that might have limited its spread requires a larger study using more comprehensive genomic data.

**Figure 2:**
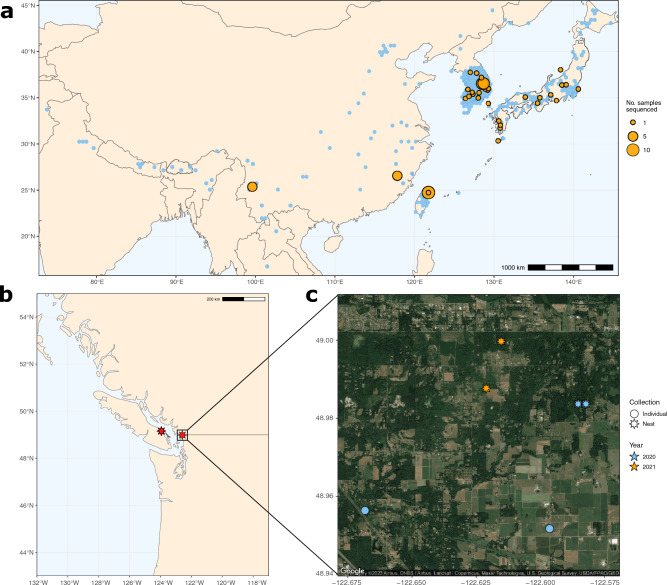
**(a)** Distribution of *V. mandarinia* observations across the species’ native range (blue), including samples sequenced as part of this project (orange). Observation data are taken from GBIF^2^. A single GBIF observation from Madhya Pradesh, India, merited closer observation due to its unusual location and was subsequently excluded as it was deemed a misidentification based on the record image; **(b)** The two localities in the Pacific Northwest in which *V. mandarinia* was discovered in 2019. **(c)** Specific locations in Blaine, WA from which samples were collected for sequencing in 2020–2021.

Here, we present the first whole-genome population genomic data from NGH samples collected in the introduced North American range and for samples from native populations in Japan, mainland China, South Korea, and Taiwan (Figs. 1 and 2). These data were sequenced with several objectives in mind: (1) to produce a much higher-resolution survey of the population genetic structure of NGH’s native range than has previously been available; (2) to confirm that the two introduced North American NGH populations have separate origins; (3) to more precisely define the locations of these origins; and (4) to characterize the degree of genomic inbreeding experienced by native and introduced NGH populations, and thereby to determine whether inbreeding depression is likely to present a major obstacle to the establishment of invasive populations of this species.

Using newly sequenced data from 114 NGH samples spanning 7 geographical regions, we generate by far the most comprehensive population genomic survey to date of any hornet species. We confirm that the samples collected in Nanaimo likely had a Japanese origin, while the origin of the Washington introduction is less clear but may be located in South Korea. We further show that samples from the Washington population exhibited strongly elevated levels of inbreeding. However, we also detect inbreeding in a number of native samples, possibly indicating that inbreeding depression alone is insufficient to prevent the persistence of NGH populations. Our results therefore emphasize the necessity of control and eradication efforts to prevent the spread of invasive populations of this species.

## Results

### Sample collection, read alignment, and variant calling

We sequenced whole genome data for 114 samples from 7 geographical regions (hereafter referred to as populations; Table 1; Fig. 2). Per-sample mean±SE mapping rate of reads was 95.8±0.004%. We mapped a total of 4.4B trimmed reads to 114 samples, with an insert size of 292.6±3.8bp, mean mapping quality of 36.7±0.2 (corresponding to an incorrect base call rate of $\sim$0.021%), and an average depth of 19.5±0.4, well above the value required for high-quality SNP calls^25^. After removing duplicates, 4.3B reads with an average insert size of 292.6±3.7bp and mapping quality 36.6±0.2 remained, with a mean depth of 18.8±0.4 for use in analyses.

**Table 1:** Per-population counts of samples used in this study.

Region	Number of samples	Native population?
Nanaimo	2	Introduced
Fujian	5	Native
Yunnan	5	Native
Japan	15	Native
South Korea	43	Native
Taiwan	13	Native
Washington	31	Introduced
Total	114	Mixed

After genotyping, the all-sites VCF included 190.5M sites, including 6.01M SNPs across all combined samples. Following filtering for depth and quality, 5.6M SNPs remained across all samples. Numbers of SNPs within each subpopulation before and after pruning for linkage disequilibrium are given in Supplementary Table S1.

For the mitochondrial super-barcoding, after QC and trimming, we analysed an alignment containing 8567 nucleotides across 103 individuals (including reference OQ836204.1 and 102 samples sequenced in this study). Five samples from Fujian, China and one from Japan were removed due to the high proportion of Ns in these sequences. To test a Chinese origin for North American NGH, the reference NGH mitogenome from Yunnan was retained in the alignment for analyses. The resulting alignment contained 422 parsimony informative sites, 42 singleton sites, and 8103 constant sites. The presence of singleton sites mainly resulted from including the single NGH reference sequence from Yunnan, China. From the 103 samples, 30 total mitochondrial haplotypes (mitotypes) were identified with two mitotypes for the five samples from Yunnan, one mitotype for the two Nanaimo samples, seven mitotypes from the 43 South Korean samples (one of which was also shared by all Washington samples), 14 mitotypes from the 14 Japanese samples, five mitotypes from the 13 Taiwanese samples, and a final remaining mitotype for reference sequence OQ836204.1 (Supplementary Table S2).

### Confirmation of separate origins for the Nanaimo and Washington introduced populations

Initial PCA using 1.03M LD-filtered SNPs grouped all samples into one of three distinct clusters along PCs 1 and 2, which together captured $\sim$25% of variation among samples (Supplementary Figure S1a). Two of these three clusters corresponded to the two mainland Chinese populations (Fujian and Yunnan), while the third included all other samples. Re-running the PCA after excluding the mainland Chinese populations allowed us to more clearly elucidate the variation among the remaining samples. These remaining samples formed four discrete clusters along PCs 1 and 2 ($\sim$18% of total variation), each representing a different population, except for a single cluster containing the Japanese and Nanaimo samples (Supplementary Figure S1b). These results held when excluding close relatives (Supplementary Figure S1c,d). PCA therefore strongly supports the possibility that the Nanaimo samples originated from Japan but does not provide the same level of support for a South Korean origin of the Washington samples.

**Figure 3:**
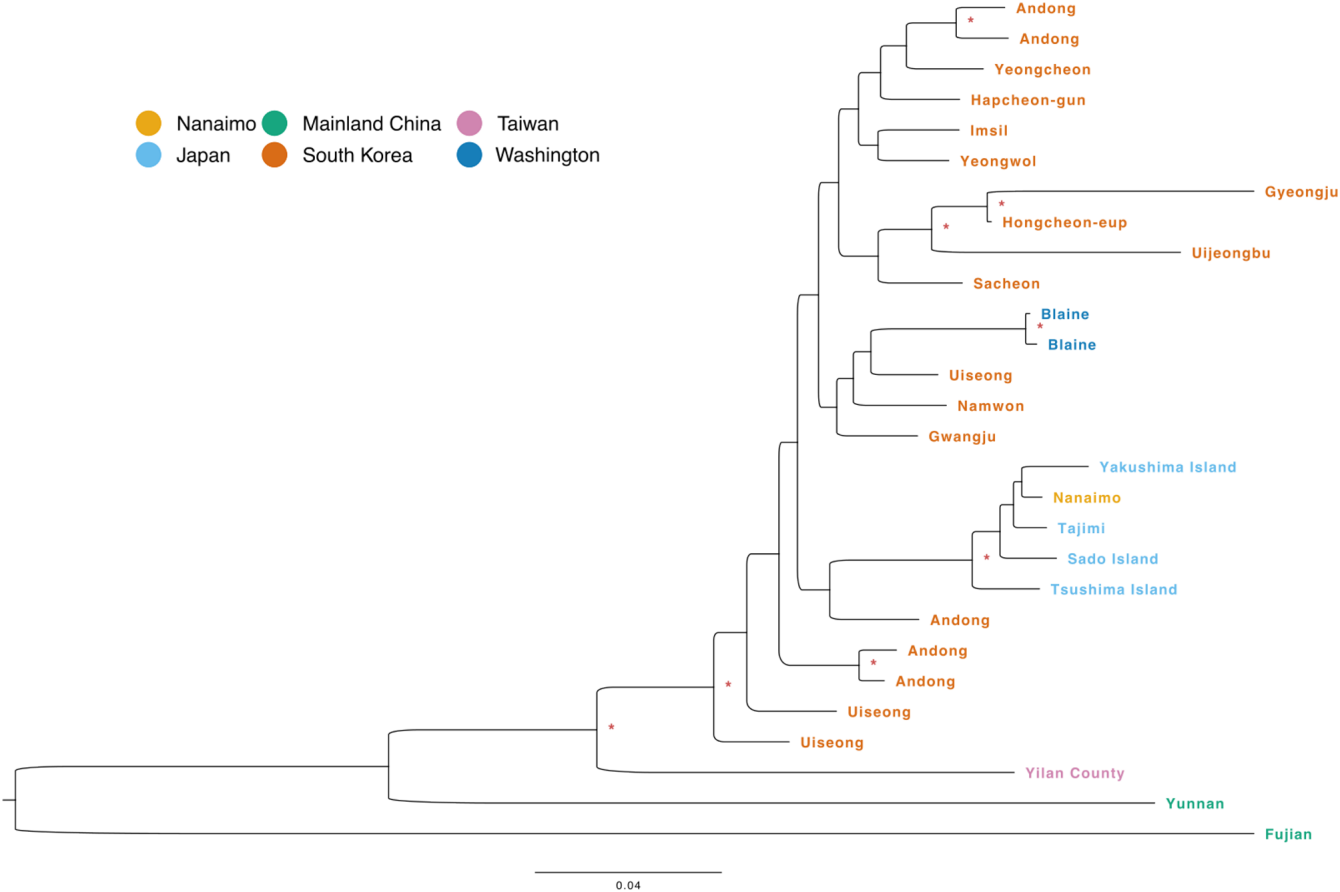
Midpoint-rooted maximum-likelihood tree using 29 unrelated *V. mandarinia* samples and 1.03M LD-pruned SNPs, calculated using the GTR+I+G4 model of nucleotide substitution. Nodes with ultrafast bootstrap support > 99% are denoted with asterisks.

Constructing a maximum-likelihood tree of distantly related individuals using SNP data supported these conclusions (Fig. 3). Most samples were separated strongly by geography in this tree, as expected given the results of the PCA, with samples from the two mainland Chinese populations and Taiwan exhibiting particularly long branches, and the Nanaimo and Japanese samples unequivocally related to one another. The South Korean samples, however, exhibited a more dispersed distribution across the tree, suggesting significant heterogeneity in this region. The Japanese and Washington samples were both nested among the Korean samples on this tree, but this was not the case when considering the TCS network and ML tree generated using mitogenomes alone, which exhibited good separation between Japanese and South Korean mitotypes (Supplementary Figure S2). All 24 samples from Washington state possessed a single mitochondrial haplotype (Vman-6), shared with 14 samples from South Korea. The two samples from Nanaimo, British Columbia possessed mitotype Vman-21. While the Nanaimo samples did not match any Japanese mitotypes exactly, they were nested in a Japanese clade with strong support in the BI and ML analyses and were different from Vman-17 by only three informative SNVs (Supplementary Figure S2). Internal branching support in both ML and BI was low in both methods and reflected in different branching orders (Supplementary Figure S2). However, in both phylogenetic trees and across all methods for quantifying branch support, South Korean, Japanese, and Taiwanese clades were resolved with high support in all cases, including the network analyses, as assessed by the number of informative sites separating the different lineages.

Using a larger SNP-based maximum-likelihood tree constructed using all samples (without pruning close relatives) produced somewhat different patterns for the Korean and Washington samples (Supplementary Figure S3). In this tree, the Washington samples stood out together with a group of South Korean samples, all of which were collected from the same nest near Andong, North Gyeongsang Province. While this might be taken as evidence that the Washington samples originated from this region, several factors speak against the likelihood of this possibility. First, Andong is not located close to any major international transport or export hubs, which makes it unlikely that hornets could have traveled directly from there to the US. Andong does export agricultural products, including rice and apples, but these goods are primarily shipped to non-US markets^26,27^. Second, the branch length between the Washington and Andong samples was much longer than that observed for example between the Nanaimo and Japanese samples. Third, the close proximity between the Washington and Andong samples on the all-samples tree was not found on the tree using only distantly related samples (Fig. 3). This indicates that the proximity of these samples was driven by some other factor, such as the fact that the all-sample tree contained a larger number of samples from these two locations than from any other single locality. Finally, while the the Washington samples shared a single mitochondrial haplotype (’Vman-6’) with the samples from Andong, this haplotype was also shared with samples from two other South Korean localities, including Yeongwul-gun, over 70 km north of Andong (Supplementary Table S2).

Compared to the Washington samples, whose exact region of origin proved challenging to discern based on our current data, the two samples found in Nanaimo exhibited high levels of genetic similarity to those sequenced from Japan. We therefore consider it highly likely that these samples originated somewhere in Japan, and as such we endeavoured to uncover the specific locality within Japan from which these samples originated. The positioning of the Nanaimo samples on the SNP-based maximum-likelihood tree (both the the full and relatedness-pruned versions) placed them as outgroups to the mainland Japanese samples, excluding samples from the islands of Sado and Tsushima (Fig. 3, Supplementary Figure S3). However, the ML tree based on mitotypes (which showed good resolution among Japanese samples) clustered the Nanaimo mitotype (Vman-21) together with mitotypes Vman-18 (Ebino), Vman-19 (Yatsushiro), Vman-20 (Kirishima) and Vman-22 (Yakushima Island), all of which are in or near southern Kyūshū, the most southerly of Japan’s four ’mainland’ islands.

### Evidence of inbreeding within native and introduced populations

Introduced populations are (at least initially) small and isolated, and as a result are expected to experience significant inbreeding^28,29^. The resultant inbreeding depression could provide a significant barrier to the establishment of invasive populations. One factor that may counteract this inbreeding depression in social insects is that a single founding female may store the sperm of multiple males in her spermatheca, although this is unlikely to be a major factor in *Vespa mandarinia* due to the very low rate of polyandry in this species^30^. Potentially more relevant, historical inbreeding in the source population may mean that that population has had time to adapt to the loss of genetic diversity, for example by purging deleterious recessive alleles^31,32^, which would reduce the severity of inbreeding depression experienced by invasive offshoots of that source population. We therefore sought to quantify the degree of inbreeding experienced by native and introduced NGH populations.

**Figure 4:**
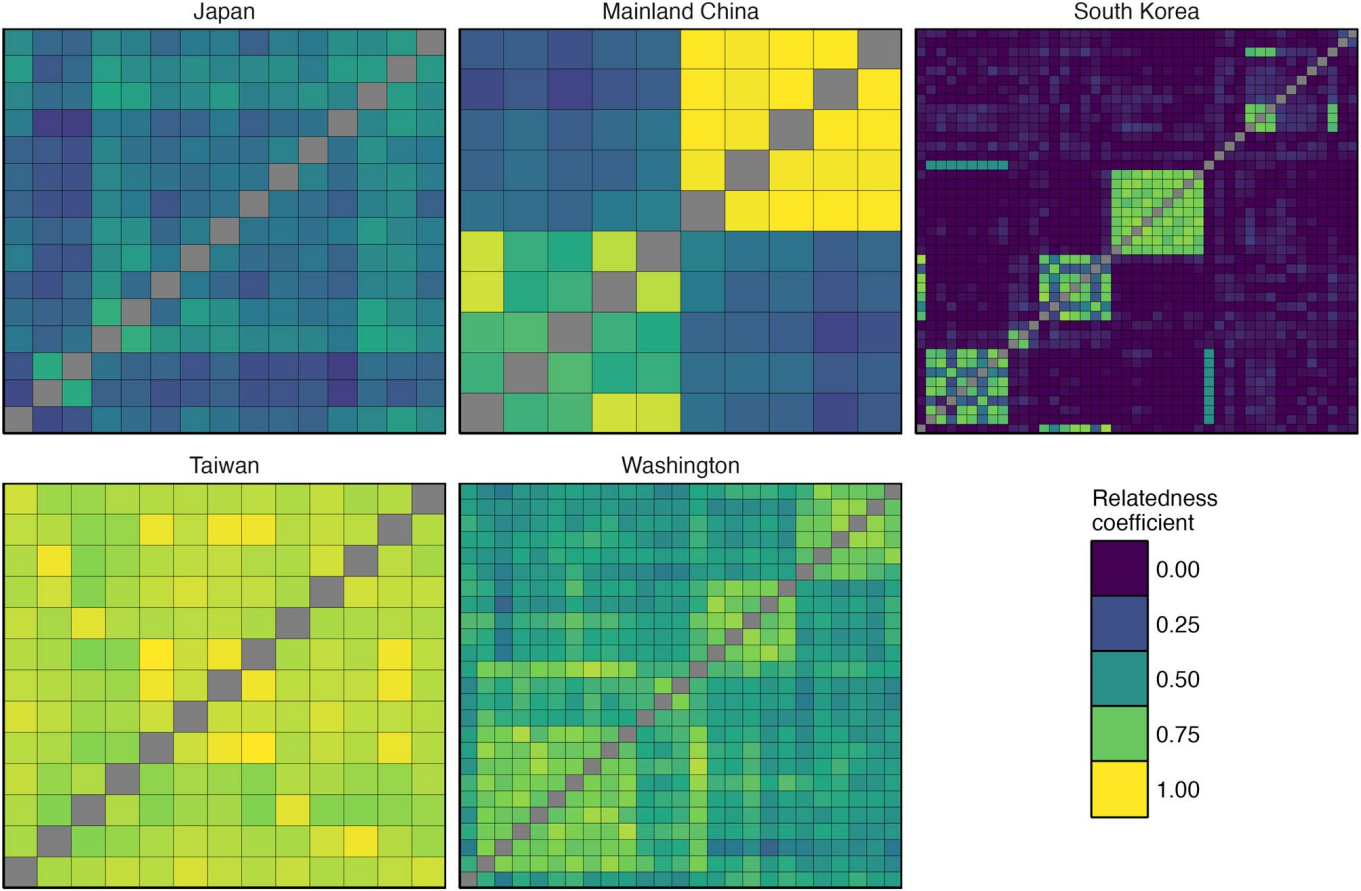
Heatmap of PolyRelatedness relatedness coefficients between pairs of female *V. mandarinia* samples within each population calculated using a diploid relatedness estimator^33^. Samples from Nanaimo, Canada are not shown, as this population included only two samples.

One way of assessing inbreeding is via the degree of relatedness between individuals within a population. Doing so using a diploid relatedness estimator^33^, we found that mainland Chinese and Korean populations exhibited a strongly bimodal distribution of relatedness values, with most individuals wholly unrelated save for a subset of closely related individuals (first-order relations collected from the same nests), as expected for an outbred population (Fig. 4). Relatedness among the Japanese samples (which did not include any within-nest replication) did not exhibit this bimodal distribution, but overall relatedness levels were higher than expected for an outbred population spanning a wide geographic area, with a mean and standard error of 0.395±0.01.

Two populations, those from Taiwan and Washington, stood out as exhibiting very high (first-order or greater) levels of relatedness between the majority of individuals, indicative of widespread inbreeding (US: 0.627±0.01; Taiwan: 0.889±0.005; Fig. 4). The extremely high relatedness values observed among the Taiwanese samples is especially surprising, given that this is a native region within which samples were collected from multiple different nests (reflected in the presence of five different mitotypes among these samples; Supplementary Table S2). These patterns remained the same when using a different relatedness estimator (Supplementary Figure S4).

Although these high levels of relatedness may indicate inbreeding, they can also arise from other factors, such as sampling structure. A more robust method of assessing the degrees of recent inbreeding within populations is by measuring the proportion of the genome, fROH, occupied by long runs of homozygosity (ROHs). fROH can be taken as an estimate of the proportion of the genome that is autozygous, i.e. identical due to inheritance from a common ancestor, and is therefore an accurate measure of inbreeding. Because ROHs will tend to be broken up over time, longer ROHs are indicative of more recent inbreeding. We measured fROH across the 6.01M SNPs among our sampled individuals at three different minimum ROH lengths (0.5, 1, and 2Mb).

**Figure 5:**
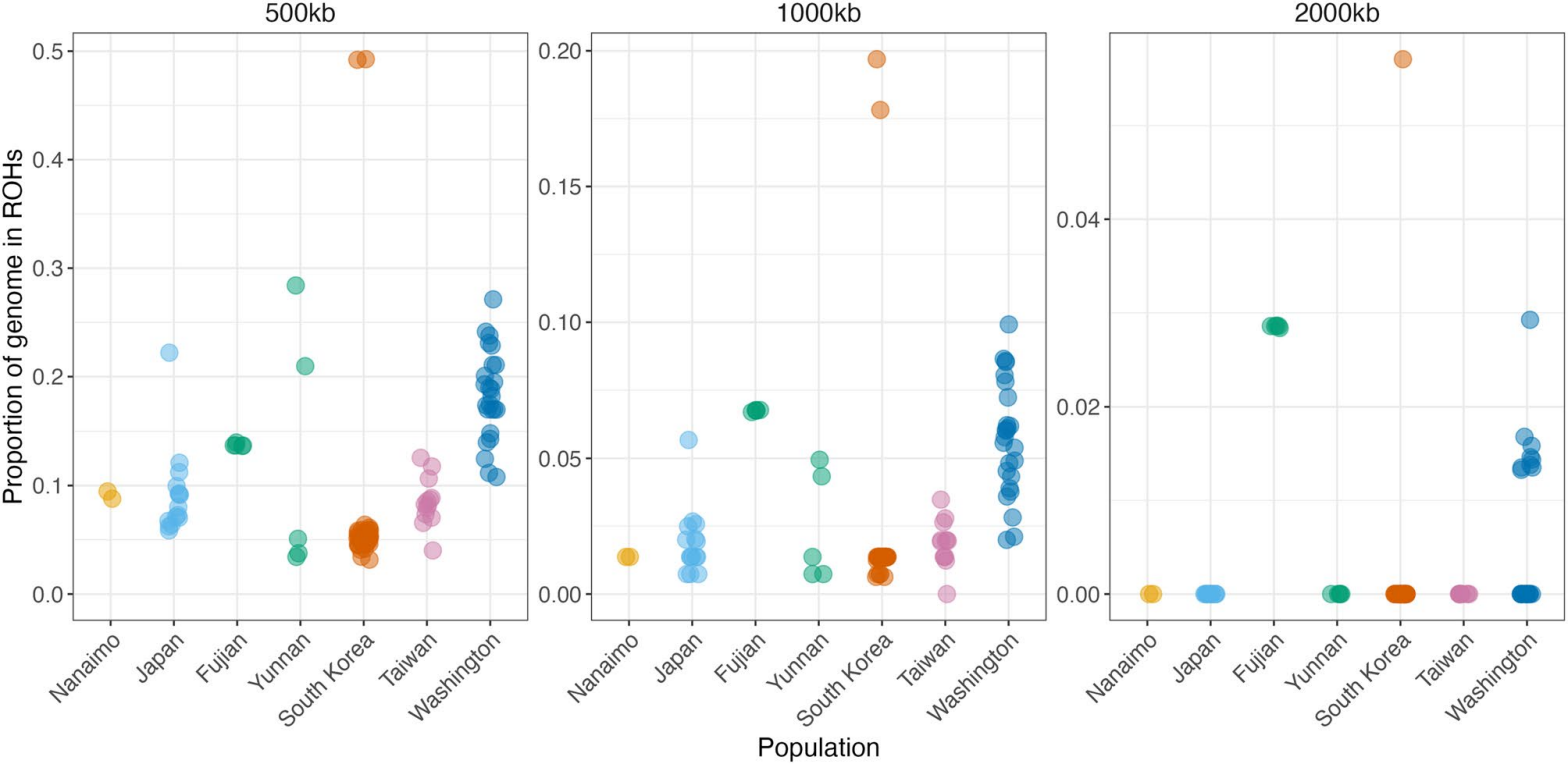
Proportion of genome occupied by long runs of homozygosity (ROHs) among *V. mandarinia* samples from different populations at three different minimum lengths of ROH. Elevated values for longer minimum ROH values indicate more recent inbreeding. Note that the scale of the y-axis varies across the three plots.

We found that, at the bottom end of ROH length (0.5 Mb), all populations exhibited fROH values greater than zero but with substantial variation within and between populations (Fig. 5). The Washington population exhibited the highest mean fROH_0.5Mb_, but a small number of individuals from other populations possessed similarly elevated values: two from Yunnan and one from Japan. Two more samples, from South Korea, exhibited extremely elevated fROH_0.5Mb_, nearly double that of the next highest sample.

At the upper end of ROH length (2 Mb), which indicates very recent inbreeding, most individuals exhibited an fROH of zero, indicating a complete lack of runs of homozygosity of this length (Fig. 5). Two populations were exceptional in this respect: all samples from Fujian and a subset of nine samples from Washington exhibited fROH_2Mb_ greater than zero. fROH_2Mb_ values among the Fujian samples were uniform, but the Washington samples fell into three groups: those with zero (fROH = 0; n = 16), intermediate (0.02 > fROH > 0; n = 8) and high (fROH > 0.02, n = 1) values of fROH_2Mb_. Of the ten females collected from the single nest in 2020, over half (5 of 9) exhibited non-zero values of fROH_2Mb_, including the single individual with fROH_2Mb_ > 0.02. Values were lower for the three nests eradicated in 2021, with 1/3, 2/6, and 1/6 of females, respectively, exhibiting non-zero fROH_2Mb_. While these sample sizes are too low to be subjected to statistical analysis, our data may reflect a reduction in the proportion of highly inbred individuals on nests found in Washington in 2021 compared to 2020.

Intriguingly, a single sample from South Korea exhibited extremely elevated fROH_2Mb_, again double that of the next-highest individual, indicative of extreme inbreeding. This sample was collected from Uijeongbu, Gyeonggi Province, which is located within a natural defile that might conceivably act as a reproductive barrier to hornets within this area and could, therefore, explain this sample’s unexpectedly high level of inbreeding.

Patterns of fROH values among populations were qualitatively similar when using only SNPs within each population (as opposed to a combined set of SNPs from all populations together), although absolute values were, as expected, much lower in this case (Supplementary Figure S5).

## Discussion

Invasive eusocial hornets represent a serious threat to agriculturally-important honey bees worldwide, as well as to native insects that play key ecological roles such as pollination and pest control. Effective management of such invasions requires that we predict their occurrences and likelihood of establishment. Here we use two short-lived introduced northern giant hornet populations in the American Pacific Northwest as exemplars for such invasions. As well as assessing the geographic origin of these populations, we assess the factors that may contribute to these populations’ ultimate eradication. We find that, while the more successful of the two introduced populations exhibited strong genomic signals of inbreeding, such signals are also present in some long-standing native populations and such inbreeding therefore likely does not present an insurmountable barrier to establishment for this species. Our results suggest that extrinsic factors, rather than inbreeding depression alone, likely contributed to the apparent failure of these introduced NGH populations to become fully established.

One factor that could feasibly have contributed to invasion failure in this case is competition with other local species, but this appears unlikely. Within its native range, NGH is an apex predator with virtually no natural enemies, and preys upon congeners and dominates use of shared resources (e.g. sap flows)^3^. Two other extrinsic factors, habitat suitability and prey availability, are also unlikely to have contributed to this failure to establish. Species distribution modelling indicates that the Pacific Northwest of North America represents a climatically suitable habitat for NGH^22,24^, which is reinforced by the initial expansion of the Washington population from one nest in 2020 to three nests in 2021, a time period across which mean temperatures and precipitation in Washington remained relatively stable^34^. Metabarcoding of larval feces from three of the NGH nests collected in Washington indicates that workers from these nests were able to successfully exploit prey species in the introduced range^35^. We also note that mate limitation may be a factor in the lack of detections over the past two seasons; in at least one native population, fewer than half of NGH females will accept a mate^3^, a characteristic whose impact may be exaggerated in a small population. Nonetheless, while we cannot conclusively rule out all extrinsic factors, evidence against a role for climate, competition or lack of prey makes it likely that concerted eradication efforts by local agencies and the public played a critical role in the ongoing extirpation of these introduced populations. Our results thus emphasize the importance of rapid response times and continued vigilance to control future such introductions.

Previous analyses of mitochondrial data revealed two separate North American origins for the Nanaimo and Washington samples, with these populations estimated as having originated from Japan and South Korea respectively^14,36^. In confirmation of this, we found that whole-genome data from the Nanaimo and Japanese samples that we sequenced exhibited strong similarity to one another while remaining genetically distinct from samples collected elsewhere in NGH’s native range. Patterns of relatedness between our samples from Washington and South Korea were more complex. Although a SNP-based maximum-likelihood tree of distantly-related individuals did place the Washington samples among those from South Korea, confidence estimates for the branching among South Korean samples was generally low (Fig. 3), and PCA placed the Washington and South Korean samples into separate clusters (Supplementary Figure S1). The single mitotype present in the Washington population was shared with a number of samples from Pungcheon-Myeon, South Korea, but this mitotype was also found in a sample from Yeongwul-gun, over 70 km distant from Pungcheon-Myeon. Although we sampled hornets from a wide variety of localities across South Korea (Fig. 2a), the Korean population exhibited a high degree of genetic diversity (Figs. 3, 4), and it is therefore possible that the Washington population originated elsewhere in South Korea. We were also unable to acquire samples for comparison from other, geographically close but harder-to-access regions such as North Korea, far north-east China, and Primorsky Krai, Russia. As such, it is likely that our dataset only captures a fraction of the total genomic variation present across NGH populations worldwide.

A striking result of both PCA and ML analysis was the relatively extreme distance of the mainland Chinese populations from each other and from all other samples. Notably, these samples were taken from populations that at one point were considered *Vespa magnifica*^37^, a designation which at present is synonymous with *V. mandarinia*^38^ but which, based on the genetic distances observed in our data, might qualify for reconsideration as a genuinely separate species or subspecies. While we sampled only a single nest each from Yunnan and Fujian, the large genetic distances of these nests between one another and with other native samples suggest that a great deal of genetic variation likely exists across the Chinese mainland. Much additional sequencing, especially across northern and central China, will be required if this variation is to be resolved.

Patterns of relatedness and of fROH (the proportion of the genome occupied by runs of homozygosity) indicate that most native populations have experienced a degree of inbreeding, with all individuals exhibiting fROH_1000kb_ greater than 0 (Fig. 5). Elevated fROH typically indicates historical inbreeding, especially in conjunction with the unusually high relatedness coefficients exhibited among individuals from the Taiwanese population. Inbreeding depression represents a significant barrier to the establishment of invasive populations^39^, but our observation of signatures of historical inbreeding in native NGH populations suggests that this species has already been exposed to a nontrivial degree of historical inbreeding, which could contribute to increased resilience against inbreeding depression (for example via the purging of deleterious recessive alleles^40^). Nonetheless, only a small number of individuals exhibited nonzero values for the longest value of fROH that we measured (Fig. 5), and almost all of these individuals were from the introduced Washington population. This does therefore indicate that this population experienced a greater degree of inbreeding than is typical in any of the native populations of this species, leaving open the possibility that inbreeding depression played a role in the eventual decline of this population. Notably, the NGH nests collected in Washington were small and appeared to be over-producing males, both of which could be indicators of inbreeding depression^19^.

Our data indicate that the Washington NGH population was likely founded by a single introduced queen: only a single mitotype was present in this population, and the high level of relatedness within this population compared to the South Korean population that represents its presumed origin is consistent with the possibility of founding by one singly-mated queen. Propagule pressure is usually considered a key factor in determining invasion success^41,42^, but our results show that for invasive hornets such as NGH a single introduction may be sufficient to begin the invasion process. This reinforces findings from the highly successful invasive *V. velutina* population in Europe, which likewise began with a single foundress^15^. The capacity of a single hornet foundress to seed an entire invasive population, even in the face of significant inbreeding, means that intercepting such foundresses in transit should be a top priority in preventing future hornet incursions in the US.

NGH is just one of several hornets that have exhibited invasive potential. At least eight *Vespa* species have been detected outside of their native ranges, of which five have been documented as establishing long-term non-native populations^4^. Of these, *V. velutina*, in addition to being the only other species for which genetic data are available, has been by far the most successful, with invasive populations established in western Europe^15^, Japan^43^ and South Korea^44^. Of particular interest as a comparison to NGH, *V. velutina*’s invasive range has spread more slowly in South Korea than in France^4,45,46^, a disparity that may be attributable to competition^47^: the only native hornet species in France is *V. crabro*, whereas in South Korea *V. velutina* must compete with five native *Vespa* species, four of which (including NGH) outcompete *V. velutina* in dominance interactions^48^. If competition with native hornets is a major factor limiting invasive success among hornets, then NGH, which is the largest and most competitively dominant of all hornet species, may be uniquely advantaged as an invader.

Although the two NGH populations introduced to the Pacific North-West in 2019 appear close to being extirpated, the threat of invasive hornets to ecosystems in North America and elsewhere remains high. In August 2023, the United States Department of Agriculture confirmed the identification of a yellow-legged hornet, *Vespa velutina*, found in Georgia, USA, and by the end of 2023 five nests had been discovered and eradicated. Preventing the establishment of this and other populations of invasive hornets requires a robust understanding of the origins of such populations and the factors that influence their probability of invasive success. The case of NGH in the Pacific Northwest should serve as an exemplar for the successful control of a introduced hornet population. Moreover, as the first whole-genome population genetic dataset available for any hornet species, our data will serve as a foundation for genetic analysis of future hornet invasions.

## Methods

### Sample collection and sequencing

NGH samples were collected from native and introduced populations from 2019-2022, except for seven samples collected in Japan in earlier years (Supplementary Table S3). For the North American introduced populations, all available samples were sequenced. Because previous analyses of mitochondrial data indicated two separate North American origins, from Japan and South Korea, respectively^14^, we collected samples from a wide range of localities in those two regions with the hope of identifying the point of origin of the introduced samples more precisely. For the remaining sampled native regions (Fujian, Yunnan, and Taiwan), samples were collected from a single locality. A summary of samples used in this paper is given in Table 1 and more complete details are provided in Supplementary Table S3.

Thoracic muscle tissue was extracted from collected samples using the MasterPure Complete DNA and RNA Purification Kit (Epicentre, Madison, WI). Library preparation was performed by Novogene Co. followed by sequencing on an Illumina NovaSeq 6000 platform with 30–50M 150-bp paired-end reads/sample.

### Read alignment and variant calling

We trimmed sequenced reads of low-quality sequences using Trimmomatic v0.39^49^. We aligned trimmed reads to the GCF_014083535.2 NGH RefSeq assembly using NextGenMap v0.5.2 with default parameters. Duplicated reads were marked and removed using GATK v4.2.2.0. GATK was then used to filter SNPs using the following filters: QD < 2.0; QUAL < 30.0; SOR > 3.0; FS > 60.0; MQ < 40.0; MQRankSum $< -12.5$; ReadPosRankSum $< -8.0$.

### Relatedness estimation

We estimated the relatedness between samples using PolyRelatedness v1.11^50^. To prepare the dataset, we filtered the raw variants using VCFtools, retaining genotype calls with a minimum quality score of 20 and removing loci with 10 percent missing data. We removed loci with a read depth of two or fewer to obtain loci suitable for parentage and relatedness analysis. We then used PLINK v2.0^51^ to filter one locus of each pair in a sliding 200-kb window using a threshold r^2^>0.1, resulting in a final set of 111,215 unlinked loci. To reduce the number of loci to a manageable amount for PolyRelatedness, we sampled every 90th locus and included those in the dataset for running the program. Finally, we ran PolyRelatedness on only female samples using a diploid relatedness estimator^33^.

### Detection of runs of homozygosity

In line with published recommendations^52^, we performed ROH analyses using SNPs un-filtered for LD. Male hornets are hemizygous and were therefore excluded from ROH analyses. All ROH estimates were generated in PLINK v2.0^51^ using the—homozyg argument with default settings except for the following:—homozyg-snp 50;—homozyg-density 60;—homozyg-gap 500; and—homozyg-window-het 1. Each ROH analysis was performed once each for minimum ROH lengths of 500 kb, 1000 kb, and 2000 kb.

For a given set of SNPs and minimum length of ROH, we first generated synthetic data for a hypothetical individual homozygous for every one of those SNPs, and then defined (ROH_max_) as the total length of ROHs that would be detected in this fully-homozygous individual. This value represented the theoretical upper bound for ROHs in any individual measured at this set of SNPs. We then defined ROH_true_ as the length of ROHs identified using PLINK in each real sample for the same set of SNPs, and defined fROH for that sample and minimum ROH length as ROH_true_/ROH_max_. In this way, for example, fROH_500kb_ for a given sample represents the length of that individual’s genome found within ROHs of 500kb or larger, relative to the maximum length of genome that could theoretically be found within such ROHs.

Estimating fROH relative to a theoretical maximum ROH for a given set of SNPs corrected for the fact that ROHs can only be detected within a certain genomic region if the density of SNPs in that region is sufficiently high. However, the choice of SNPs used may introduce biases, for example when a site is fixed for two different alleles across two populations, such that it is polymorphic when considered across the two populations but is monomorphic (and therefore always homozygous) when looking within a single population. To account for this possibility, we generated fROH values in each population twice: once using the full set of SNPs discovered across all populations, and again for each population using only SNPs that were polymorphic within that population.

### Principal component analyses and maximum-likelihood trees

To determine the genetic structure of populations in each species across their range, we used principal component analyses (PCAs) and maximum-likelihood (ML) trees. Prior to these analyses, SNPs were filtered to exclude sites in strong linkage disequilibrium (LD) with one another. We used PLINK v2.0^51^ to remove SNPs with pairwise r^2^>0.8 within sliding windows of 50 SNPs, with a 5-SNP increment between windows, a standard parameterization^53^.

We performed principal component analysis using the same linkage-pruned datasets in R v4.1.2^54^ using the snpgdsPCA() function, part of the package *SNPRelate* v1.26.0^55^. Because the initial PCA showed the two mainland Chinese populations to be highly distinct from all other populations, such that it was not possible to resolve the clustering of samples among and within the other populations, we also generated additional PCAs without the mainland Chinese samples.

Some of the samples in our dataset were closely related to one another, for example because they were sampled from the same nest. Our initial testing found that including many close relatives with very short branch lengths resulted in ML trees that were very crowded and inflated the branch lengths to clusters of close relatives. We, therefore, pruned our data of closely related individuals before the construction of ML trees using NAToRA^56^ together with the relatedness values generated using PolyRelatedness. Using a relatedness cutoff of 0.33, the harmonic mean of 0.5 and 0.2 (relatedness values denoting first- and second-order relatives, respectively), we pruned our dataset down to 29 distantly-related individuals.

We constructed ML trees using relatedness- and LD-filtered SNP data in R v4.1.2^54^. Data were read into R using *vcfR* v1.14.0^57^ and converted to ML trees using *ape* v5.7^58^ and *phangorn* v2.11.1. To speed computation, trees were generated using a subset of 10,000 SNPs. Model suitability was assessed using the modelTest() function and a GTR+G(4)+I nucleotide substitution model was selected as optimal based on BIC values. Bootstrap values were obtained using 1000 ultrafast bootstrap replicates^59^, as implemented by phangorn. Tree files were converted to Newick format and plotted using FigTree v.1.4.4^60^. For readability, bootstrap values in the figures were binarized, with nodes with bootstrap support > 99% denoted with an asterisk (*).

### Mitochondrial super-barcoding

To further examine the origins and genetic diversity of introduced NGH to Washington state, we applied a super barcode approach^61,62^ utilizing mitogenomic data from the whole genome shotgun data to quantify the number of foundresses involved with the initial introduction. To do this, reads from the mitogenome were extracted from the whole genome data by comparison to the* V. mandarinia* reference mitogenome NC_050197.1. The reads were then assembled into consensus sequences for each individual using BCFtools 1.8^63^, with missing alleles set to N. To ensure high- quality SNV calls were used in haplotype identification, regions in the consensus sequences with less than 100x mean read depth were removed. The alignment was further trimmed by removing first individuals with more than 0.4% Ns (indicative of low individual coverage and/or poor-quality template DNA) and then all loci across all remaining individuals that contained any Ns or ambiguous bases. Proceeding trimming and after each trimming step, alignments were generated using MAFFT 7.450^64,65^. Annotated NGH mitogenome reference OQ836204.1 was included through the above steps to assess the genic location of SNVs and ensure that the data set generated in this study was not frameshifted in relation to the reference due to the above trimming steps. The trimmed sequences were also back- checked by mapping the trimmed sequences to the untrimmed reference. The trimmed and checked alignment was then used to generate a Maximum Likelihood (ML) phylogenetic tree using IQ-TREE^66^ with default settings, model selection using ModelFinder^67^ and clade support assessed using ultrafast bootstrapping^59^ as well as the nonparametric SH-aLRT^68–70^. Based on these results, a Bayesian inference (BI) tree was resolved using our samples from Yunnan as an outgroup, given their genetic distance from the remaining samples and their closest match to *V. magnifica* in BLASTn. The program MrBayes 3.2.6^71^ was employed to conduct Bayesian inference and was run with a general time reversible + invariable sites + gamma rate variation among sites substitution and rate variation model, four gamma categories, six heated chains, a chain length of 1,100,000, a subsampling frequency of 200, a burn-in of 100,000, and an unconstrained branch length prior. Finally, a TCS statistical parsimony^72^ network was generated from the alignment using PopART 1.7^73^. Haplotype calls and sequence characterizations were implemented in DNAsp 6.12.03^74^.

### Supplementary Information

Supplementary Information 1.

Supplementary Information 2.

## Data Availability

Raw sequencing data are available at the Sequence Read Archive (SRA) under BioProject PRJNA1069611.
