# Supplementary material for: Population genomics of the invasive Northern Giant Hornet Vespa mandarinia in North America and across its native range
Source: Sci Rep. 2024 May 11;14:10803. doi: 10.1038/s41598-024-61534-0 (PMC11088652; doi:10.1038/s41598-024-61534-0)

**Figure S1. (a & b)** PCAs of all sequenced native and invasive *V. mandarinia* samples using LD-pruned SNPs; **(c & d)** PCAs using the same SNPs, after exclusion of closely-related samples. **(a & c)** 1.03M SNPs among all samples; **(b & d)** 0.76M SNPs after excluding samples from mainland China. Samples from most populations are very tightly clustered, resulting in overlapping points; accordingly, a small amount of offset has been applied to points' positions to improve visibility of individual samples.

**d**

**c**

**
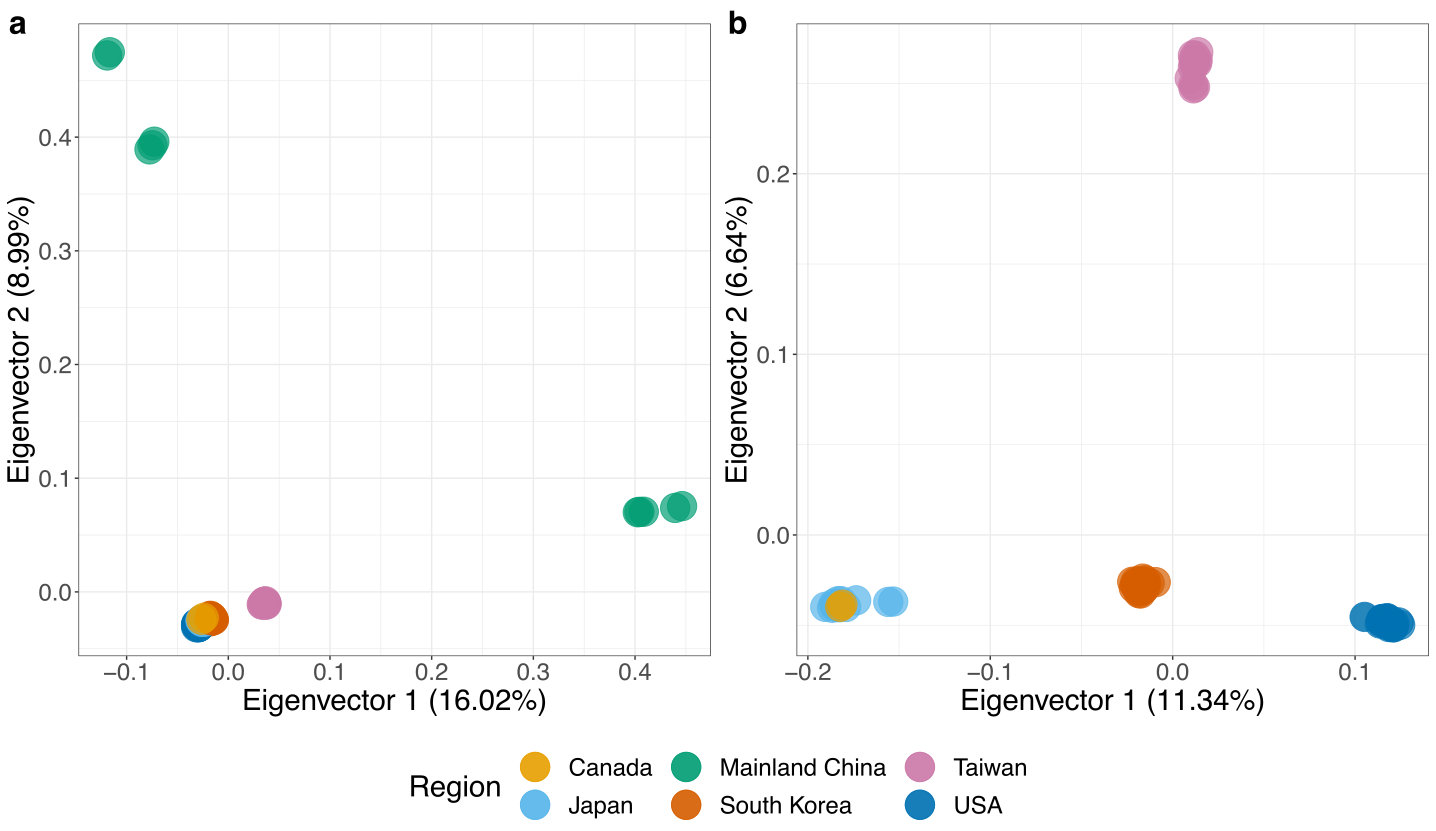
**

**Figure S2.** Mitogenome population analyses for NGH samples sequenced in this study. **(a)** A TCS network indicating the number of informative nucleotide differences (SNVs) between haplotypes and groups of haplotypes. Clusters are color coded by origin and numbers refer to the ‘Vman’ haplotype numbers in Table S2. **(b)** An ML tree resolved from a mitogenome alignment of 103 *V. mandarina* samples. Color coding, numbering, and the sequences used are the same as in subfigure **a**. Support values are SH-aLRT/ultrafast bootstrap/posterior probability and are indicated for pertinent branches.

**
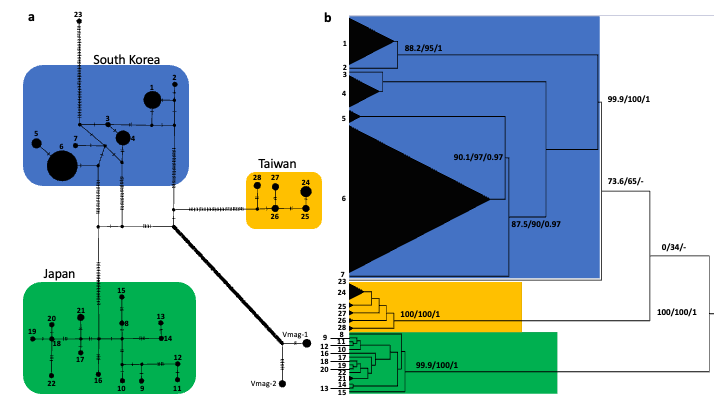
**

**Figure S3.** Maximum-likelihood tree using 106 female *V. mandarinia* samples and 1.03M LD-pruned SNPs, calculated using the GTR+I+G4 model of nucleotide substitution. Tree is rooted using the most distant population, Fujian. Nodes with ultrafast bootstrap support > 99\% are denoted with asterisks.

**Figure S4.** Heatmap of PolyRelatedness relatedness coefficients between pairs of *V. mandarinia* samples within each population calculated using the Thomas (2010) estimator (denoted Poly11 in Polyrelatedness).


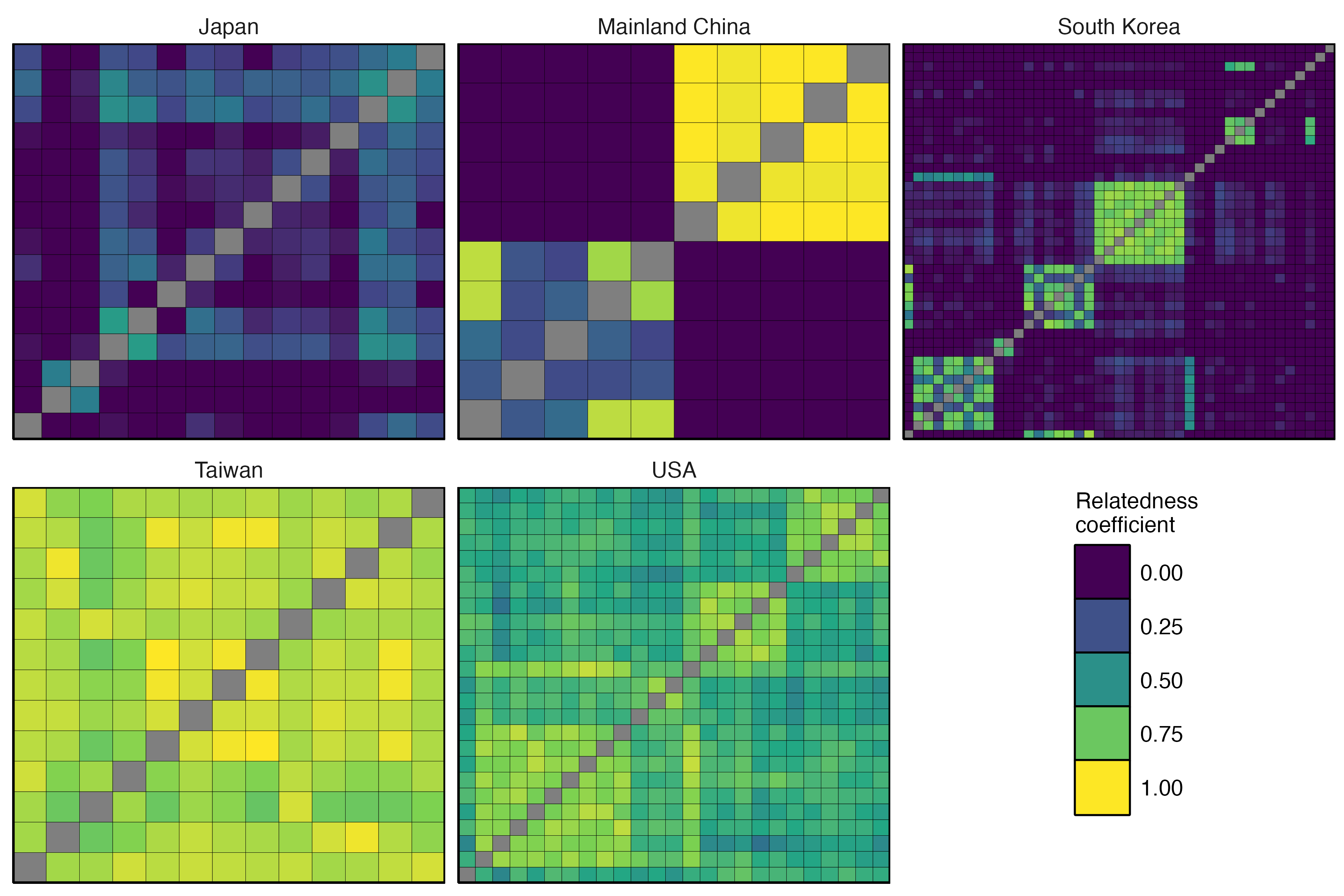


**Figure S5.** Proportion of genome occupied by runs of homozygosity among *V. mandarinia* samples from different populations, using only SNPs present within each population.


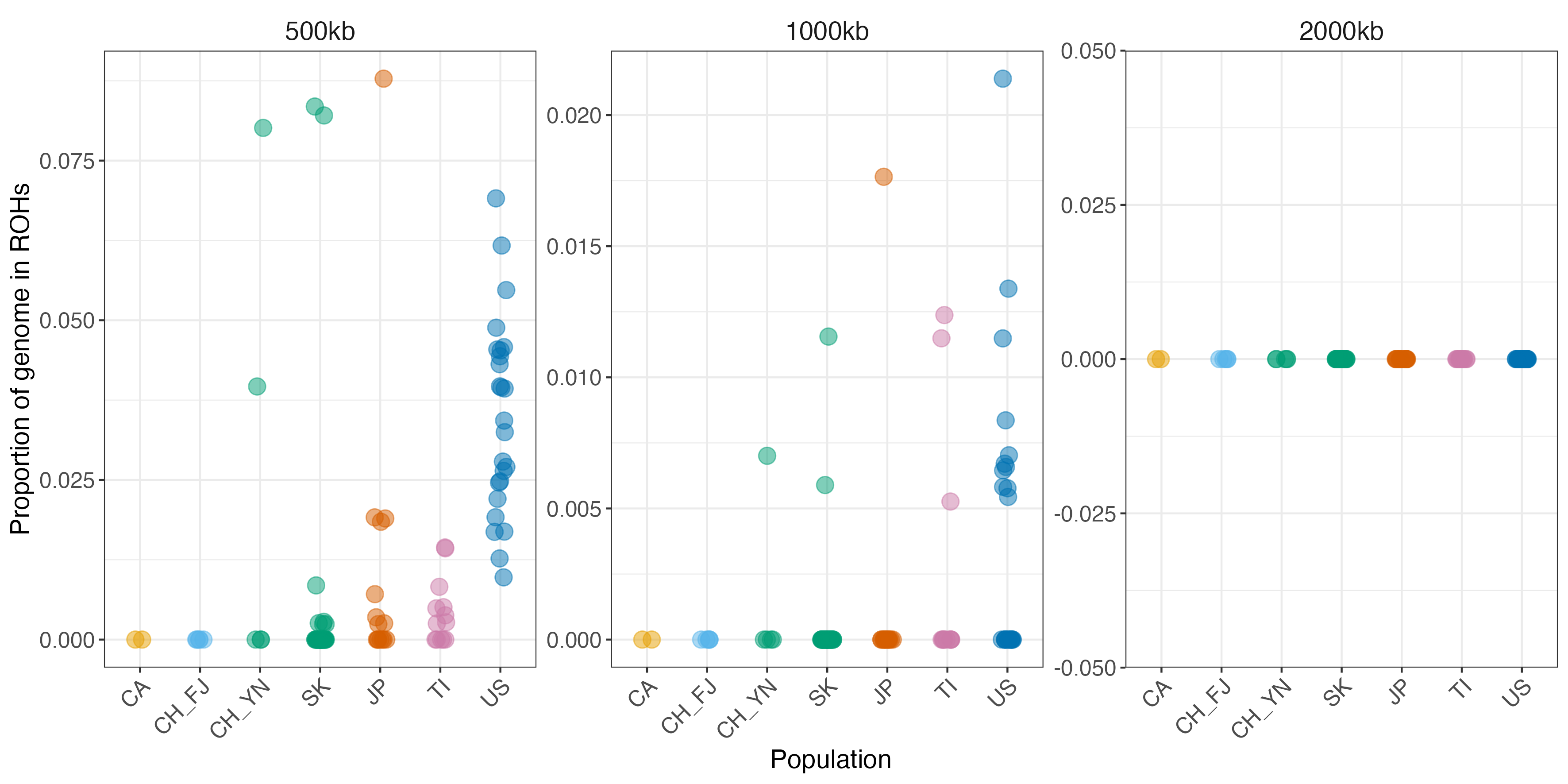

Supplement: Supplementary file 1 — Supplementary Information 1. [file 41598_2024_61534_MOESM1_ESM.docx]
